# Comparative Proteomic Analysis Reveals Proteins Putatively Involved in Toxin Biosynthesis in the Marine Dinoflagellate *Alexandrium catenella*

**DOI:** 10.3390/md11010213

**Published:** 2013-01-22

**Authors:** Da-Zhi Wang, Yue Gao, Lin Lin, Hua-Sheng Hong

**Affiliations:** State Key Laboratory of Marine Environmental Science, College of the Environment and Ecology, Xiamen University, Xiamen 361005, China; E-Mails: gaoyue9458@sina.com (Y.G.); linlin1982@xmu.edu.cn (L.L.); hshong@xmu.edu.cn (H.-S.H.)

**Keywords:** marine dinoflagellates, *Alexandrium catenella*, paralytic shellfish toxins, cell cycle, toxin biosynthesis, proteomics, mass spectrometry

## Abstract

*Alexandrium* is a neurotoxin-producing dinoflagellate genus resulting in paralytic shellfish poisonings around the world. However, little is known about the toxin biosynthesis mechanism in *Alexandrium*. This study compared protein profiles of *A. catenella *collected at different toxin biosynthesis stages (non-toxin synthesis, initial toxin synthesis and toxin synthesizing) coupled with the cell cycle, and identified differentially expressed proteins using 2-DE and MALDI-TOF-TOF mass spectrometry. The results showed that toxin biosynthesis of *A. catenella* occurred within a defined time frame in the G1 phase of the cell cycle. Proteomic analysis indicated that 102 protein spots altered significantly in abundance (*P* < 0.05), and 53 proteins were identified using database searching. These proteins were involved in a variety of biological processes, *i.e.*, protein modification and biosynthesis, metabolism, cell division, oxidative stress, transport, signal transduction, and translation. Among them, nine proteins with known functions in paralytic shellfish toxin-producing cyanobacteria, *i.e.*, methionine *S*-adenosyltransferase, chloroplast ferredoxin-NADP+ reductase, *S*-adenosylhomocysteinase, adenosylhomocysteinase, ornithine carbamoyltransferase, inorganic pyrophosphatase, sulfotransferase (similar to), alcohol dehydrogenase and arginine deiminase, varied significantly at different toxin biosynthesis stages and formed an interaction network, indicating that they might be involved in toxin biosynthesis in *A. catenella*. This study is the first step in the dissection of the behavior of the *A. catenella *proteome during different toxin biosynthesis stages and provides new insights into toxin biosynthesis in dinoflagellates.

## 1. Introduction

The dinoflagellate genus *Alexandrium* is one of the major harmful algal bloom genera along the coastal regions of the world [1]. Many *Alexandrium* species are known to produce paralytic shellfish toxins (PSTs), a group of neurotoxic alkaloids which selectively block voltage-gated Na^+^ channels in excitable cells, thereby affecting neural impulse generation and resulting in paralytic shellfish poisoning (PSP) [2]. Currently, there is no antidote for PSP and more than 2000 cases of human poisonings occur per year on a global basis, with a mortality rate of 15% [3]. Due to the public health and ecosystem impacts of toxic *Alexandrium* blooms, the genus has been extensively studied. Moreover, PSTs are highly effective compounds for relieving withdrawal symptoms in opiate-addicted patients and have potential clinical uses [4]. Much effort has been devoted to the toxin producing physiology of different *Alexandrium* species and several toxin biosynthesis pathways are postulated [1,5,6,7,8,9,10,11,12,13,14]. However, little is known concerning the toxin biosynthesis mechanismsin *Alexandrium*.

Studies show that toxin biosynthesis is regulated by genes in dinoflagellates, and at least the interconverting enzymes are encoded by nuclear genes [15]. In *A. fundyense*, PST production occurs during a discrete time period localized in the early G1 phase of the cell cycle [8], and three genes encoding *S*-adenosylhomocysteine hydrolase (SAHH), methionine aminopeptidase (MAP), and a histone-like protein (HLP) are proposed to be involved in toxin biosynthesis [10]. In *A. catenella*, SAHH and Map are related to toxin synthesis indirectly while *S*-adenosylmethionine (SAM) is directly involved in toxin biosynthesis [16]. A recent study using a microarray-based comparison of toxic and non-toxic strains of *A. minutum* reveals several unique genes in the toxic *A. minutum*. However, their roles in toxin biosynthesis are unclear [17,18]. Overall, the identification of toxin-related genes or proteins has made a substantial contribution to understanding the molecular basis of PST biosynthesis, but the mechanisms regulating toxin biosynthesis are still unclear in dinoflagellates.

Recently, the complete sequence of the PST gene cluster (*sxt*) has been revealed in a cyanobacterium, *Cylindrospermopsis raciborskii *T3 [19], and preliminary sequence similarity analyses predicate the putative functions and origins of 26 PST genes. On this basis, the draft genome assembly of toxic *Anabaena circinalis* ACBU02 and its nontoxic sister *Anabaena circinalis* ACFR02 is compared, and 13 genes unique to PST-producing *A. circinalis* are identified [20]. This study also demonstrates that the assembly of PST genes in ACBU02 is involved in multiple horizontal gene transfer events from different sources, followed presumably by coordination of the expression of foreign and native genes in the common ancestor of toxic cyanobacteria. In dinoflagellates, the transcripts of *sxt*A, the unique starting gene of PST synthesis, are found to have the same domain structure as the cyanobacterial *sxt*A gene [15]. However, so far, no cyanobacterial PST genes have been detected in toxic *A. minutum* [17], indicating that the PST genes in dinoflagellates might be different from their cyanobacterial counterparts [21]. 

Proteins are the “workhorse” molecules of life, participating in essentially every structure and activity of life. Proteomics is a global technique that provides effective strategies and tools for profiling and identifying the proteins of various organisms, including dinoflagellates [22,23,24,25]. A “toxicity biomarker” is identified from toxic *Alexandrium *species using the proteomic approach although the function of this biomarker is still unclear [26]. A recent study involving the proteomic-based comparison of a toxicity-lost mutant and the wild-type of *A. catenella *reveals differentially expressed proteins that might be responsible for the loss of toxicity in the mutant *A. catenella* [27]*. *Thus, it is reasonable to expect that the study of proteins should help to uncover the toxin biosynthesis mechanisms in dinoflagellates.

In one of our previous studies, we found that toxin production of *A. catenella *is a cell cycle-dependent activity and toxin is synthesized in a defined interval within the G1 phase [28], suggesting that the proteins or enzymes involved in toxin biosynthesis might be expressed in a defined interval. In this study, we compared the protein profiles of synchronized *A. catenella* cells collected at different toxin biosynthesis stages, and identified differentially expressed proteins using 2-DE and MALDI-TOF-TOF mass spectrometry. A total of 53 proteins which altered significantly in abundance (*P* < 0.05) at the different toxin biosynthesis stages were identified, and these proteins were involved in various biological processes. Among them, nine proteins, with known functions in PST-producing cyanobacteria, formed an interaction network, and were putatively involved in toxin biosynthesis in *A. catenella*.

## 2. Results and Discussion

### 2.1. Diel Phasing of the Cell Cycle of *A. catenella*

**Figure 1:**
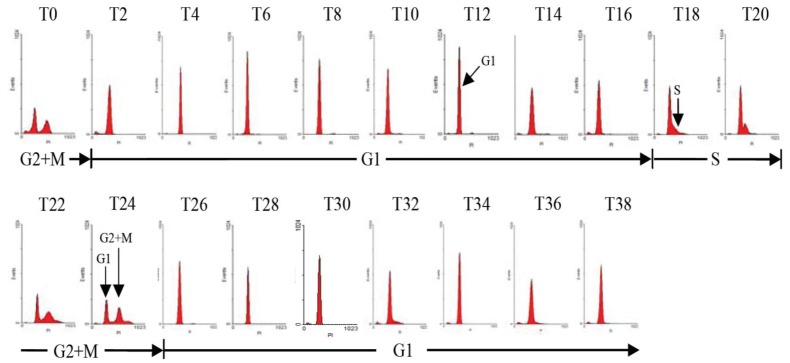
Diel cell-cycle phasing of *A. catenella* after synchronous growth in a 14:10 h light/dark cycle. Cell cycle distribution was determined using flow cytometry of DNA-stained cells harvested at 2 h intervals. The *x*/axis represents the relative amount of DNA and the *y*/axis the number of cells in a sample containing a particular amount ofDNA.

To determine the diel phasing of the cell-cycle, *A. catenella *cells were harvested throughout a diel cycle, stained using propidium iodide, and then analyzed for DNA fluorescence using flow cytometry. The representative DNA histograms of *A. catenella *within one diel cycle are shown in Figure 1. Flow cytometric analysis showed that *A. catenella* grown in the nutrient replete condition presented discrete G1, S, G2 + M phases during a 24 h circadian day. After mitosis, cells were primarily maintained in the G1 phase for about 16 h (T2–T18) after the onset of the light cycle. S phase entry started approximately 4 h after the onset of dark cycle and lasted for about 4 h (T18–T22). The percentage of G2 + M phase cells was consistently maximal approximately 10 h after the onset of dark and lasted for less than 4 h (T22–T26). Namely, the vast majority of cells (about 75%) in the G2 + M phase occurred at the point of dark/light transition (T24). Subsequently, the cells completed division within 2 h (T24–T26) of the onset of light. Then the cells entered the G1 phase and prepared for the next cell division cycle.

### 2.2. Diel Variations of Cell Density and Toxin Content of *A. catenella*

Diel variation of the cell density of *A. catenella* is shown in 2A. The initial ce ll density was approximately 3500 ± 180 cells/mL, but after the first cell cycle run, the cell density increased to 6180 ± 327 cells/mL, and about 77% of the cells completed mitosis. When the cells entered the second cell cycle run, cell density remained constant for the first 20 h and cell division began 8 h after the cells entered the dark phase and lasted for 2–4 h. The cell density increased from 6180 ± 327 to 11,470 ± 598 cells/mL at the end of the second cell cycle run, and about 86% of the cells completed mitosis. 

**Figure 2:**
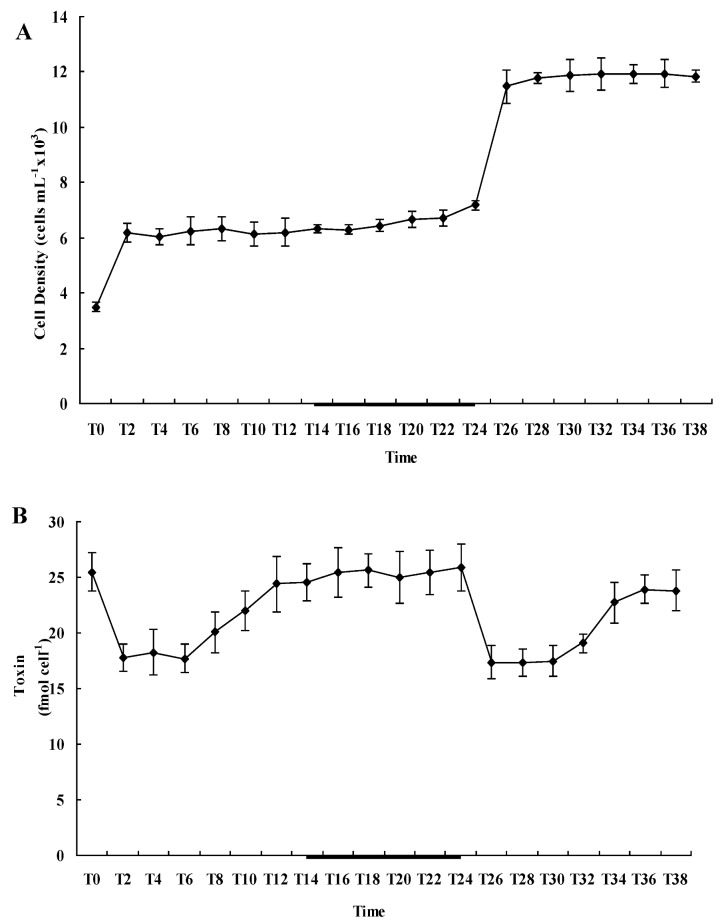
Diel variation of (**A**) cell density and (**B**) toxin content within one cell cycle in *A. catenella* after synchronous growth in a 14:10 h light/dark cycle (dark period indicated by the black segment of the X coordinate, adapted from Gao *et al.*, 2012 [28]). Cell density was measured at 2 h intervals (Error bars denote ± SD, *n* = 3).

The cellular toxin content (Qt) variation during the cell cycle is shown in Figure 2B. Apart from C1, C2, GTX2 and GTX3, no other PSP toxin derivatives were detected in *A. catenella*. When the cells completed mitotic division, the Qt decreased sharply from 25.5 ± 1.71 fM/cell to 17.8 ± 1.2 fM/cell within 2 h and then remained relatively constant for 4 h. After that, the Qt increased to 24.4 ± 2.5 fM/cell within 6 h and remained constant for the next 12 h. Similarly, after the second cell division run, the Qt reduced obviously from 25.9 ± 2.1 fM/cell to 17.4 ± 1.5 fM/cell within 2 h and maintained relative constant for 4 h. Then the Qt increased to 23.9 ± 1.3 fM/cell within 6 h and remained constant before the next mitosis. This result indicated that toxin biosynthesis was not a continuous event in *A. catenella* and occurred within a defined time frame during the G1 phase, which is consistent with the result reported in *A. fundyens * [8]. 

### 2.3. Protein Identification and Variation at Different Toxin Biosynthesis Stages

**Figure 3:**
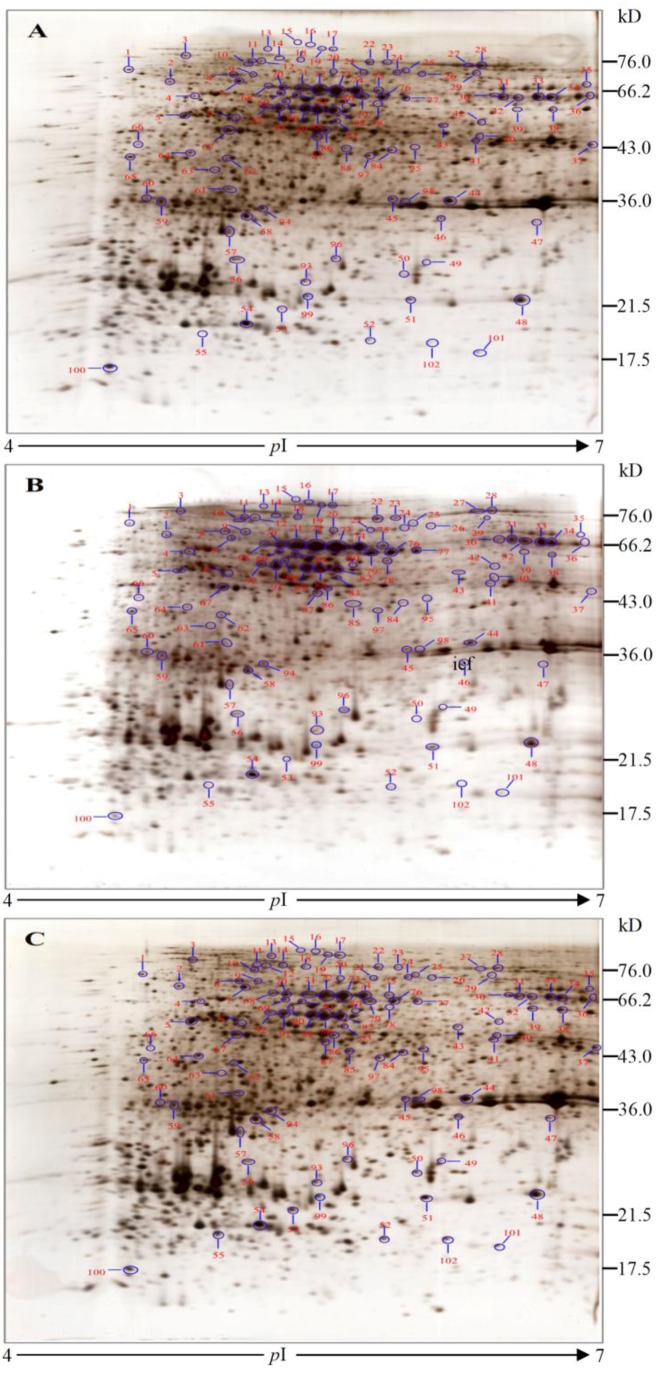
2-DE protein profiles of *A. catenella* at different toxin biosynthesis stages. (**A**) T24, non-toxin synthesis stage; (**B**) T28, initial toxin synthesis stage; (**C**) T34, toxin synthesizing stage.

Protein profiles of *A. catenella *at the different toxin biosynthesis stages were analyzed using 2-DE, and the representative 2-DE images of the different time points (T24, T28 and T34) are shown in Figure 3. The 2-DE gels of T24 (non-toxin synthesis stage) were selected as the reference gels. For each biological replicate, three technical replicates were conducted at the same time and the reproducibility of this protocol was more than 95%. 

Based on auto-matching using gel analysis software (Image Master 2D Platinum 6.0, GE Healthcare) and a manual quality check of the detected spots, 2244 protein spots were matched preferably in the three group gels. A total of 102 exhibited statistically significant alterations (*P* < 0.05) and the variations in abundance were more than 2 fold. All the altered spots were submitted for identification using MALDI-TOF-TOF MS and searched in the Swiss-Prot, NCBInr protein and dinoflagellate polypeptide databases. Positive identifications were obtained for 91 spots, leaving two unknown protein spots (spots 21 and 44) and nine negative identifications (spots 30, 43, 51, 80, 86, 99, 100, 101 and 102). Only the data for the 91 positive spots were used for further analysis, and the details of NCBI ID number, theoretical *p*I value, theoretical molecular weight, protein score, protein score C.I.%, as well as the average relative change at each time point are listed in Supplemental Table S1.

**Figure 4:**
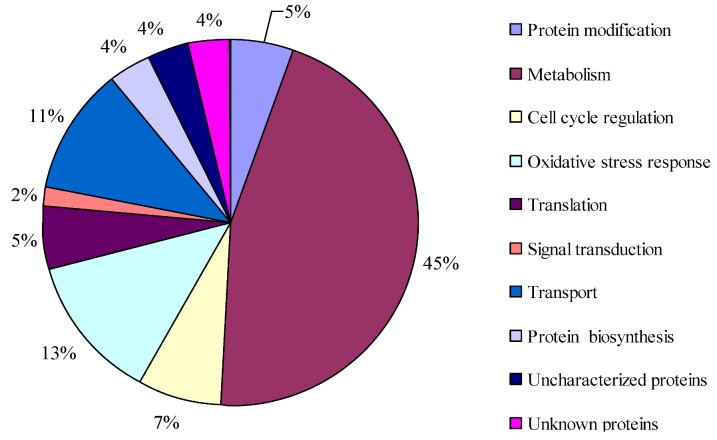
Functional classification of differentially expressed proteins of *A. catenella* at different toxin biosynthesis stages.

Based on KOG classification, 51 proteins out of the 91 protein spots were assigned into eight functional groups, *i.e.*, the protein modification, metabolism, cell cycle regulation, oxidative stress response, translation, signal transduction, transport, and protein biosynthesis (Figure 4). A large number of proteins (45%) involved in various metabolisms were altered significantly in abundance: four proteins related to the one-carbon metabolic process were identified, namely serine hydroxymethyltransferase (spots 8 and 42), methionine (*S*) adenosyltransferase (spots 31, 32, 33, 34, 36), adenosylhomocysteinase (AdoHcy, spots 76 and 79), *S*-adenosylhomocysteinase (SAH, spot 77). Among them, serine hydroxymethyltransferase was down-regulated at T28 and then recovered to the normal level at T34. In contrast, the other proteins were up-regulated or invariable at T28, and down-regulated at T34. Three proteins, phosphoglycerate kinase (spot 19), fructose-bisphosphatealdolase (spot 67) and enolase 2 (spot 69) were involved in glycolysis. The abundance of phosphoglycerate kinase increased from T28 to T34. Concurrently, fructose-bisphosphatealdolase and enolase 2 were up-regulated at T28 and recovered to the normal values at T34. Seven proteins were involved in photosynthesis. Among them, chloroplast ferredoxin-NADP+ reductase (FNR, spot 37), plastid oxygen-evolving enhancer 1-2 precursor (spots 45), light-harvesting polyprotein precursor (spot 56), ribulose bisphosphate carboxylase oxygenase large subunit (spots 72, 73, 74) and chlorophyll A–C binding protein (spot 93) were down-regulated at T28 then recovered to the normal levels at T34. Photosystem I assembly protein ycf1 (spot 49) and ribulose bisphosphate carboxylase oxygenase small subunit (spot 70) were down-regulated at T28 but were up-regulated more than 2 fold at T34. Three enzymes, inorganic pyrophosphatase (PPi, spot 55), arginine deiminase (ADI, spot 78), and similar to sulfotransferase (SULT, spot 94) were identified; and PPi varied little at T28 but was up-regulated more than 11 fold at T34. The abundance of ADI increased 3 fold at T28 and decreased obviously at T34. SULT was down-regulated at T28 then recovered to the normal level at T34. 

Three proteins, chaperone protein DnaK (spot 1), Hsp70-type chaperone (spot 3) and chaperonin GroEL (spots 7 and 12) were down-regulated at T28 and up-regulated at T34. Three proteins were possibly involved in cell cycle regulation, *i.e.*, cell division protein FtsH (spot 9), formamidopyrimidine-DNA glycosylase (FPG, spots 38 and 39), DNA damage checkpoint protein rad24/17 (spots 62 and 64). The abundance of FPG decreased at T28 and was up-regulated more than 4 fold at T34. Several proteins related to the oxidative stress response were also identified. Heat shock protein 70 (spots 10, 11, 14, 18) involved in the folding and unfolding of other proteins was up-regulated from T28 to T34. DNA-binding stress protein (spot 53), ketol-acid reductoisomerase (spot 84), alcohol dehydrogenase (ADH, spot 95) and RNA-binding S1 domain protein (spot 96) were down-regulated at T28, but were up-regulated significantly at T34. Nine isoforms of luciferin-binding protein (spots 20, 22–29) were identified and they presented different expression patterns at different toxin biosynthesis stages. Formyltetrahydrofolate deformylase (spot 98), putatively involved in the *de novo* inosine monophosphate biosynthetic process, was slightly down-regulated at T28 and remarkably up-regulated at T34.

In addition to the above proteins, others involved in translation, signal transduction, transport, and protein biosynthesis were also identified in this study, *i.e.*, elongation factor P (spots 61 and 63), hypothetical protein PP_4544 (spot 5), UDP-*N*-acetylglucosaminedolichyl phosphate (spot 58) and actin protein (spot 85), presented differentially expressing patterns during the different toxin biosynthesis stages.

### 2.4. Proteins Putatively Involved in Toxin Biosynthesis

Much effort has been devoted to elucidate biosynthesis genes and enzymes of PSTs in dinoflagellates, and several enzymes involved in PST biosynthesis, *i.e.*, *S*-adenosylhomocysteine hydrolysis, aminotransferase, methionine aminopeptidase, Na(+)-dependent transporter, *O*-carbamoyltransferase and sulfotransferase are reported in some dinoflagellate species [10,14,19,29,30]. Recently the PST gene cluster (*sxt*) was revealed in a cyanobacterial species, *C. raciborskii* T3 [19], which encodes 30 catalytic functions to 26 proteins involved in toxin biosynthesis. Further comparison of the draft genome assembly of the saxitoxin-producing *A. circinalis* ACBU02 and its nontoxic sister *A. circinalis* ACFR02, reveals 13 genes unique to PST producing cyanobacteria [20]. This study identified 53 differentially expressed proteins at different toxin biosynthesis stages. Among them, nine proteins, *i.e.*, methionine *S*-adenosyltransferase (MAT), SAH, AdoHcy, PPi, ornithine carbamoyltransferase (OTC), SULT, ADH, FNR and ADI, were altered significantly in abundance at different toxin biosynthesis stages (Table 1), and are reported to be involved in PST biosynthesis in cyanobacteria [19]. AdoHcy and SAH have been identified in dinoflagellates and are two important enzymes catalyzing or converting *S*-adenosylhomocysteine to homocystein and adenosine [10,21,31,32], which play vital roles in the metabolism of amino acid and nucleic acid. Homocystein can be recycled into methionine, a primary precursor in the first step of PST biosynthesis. The significant up-regulation of these two enzymes at the initial toxin biosynthesis stage (T28) indicated that more *S*-adenosylhomocysteine might be converted to homocystein which then provided methionine for subsequent toxin synthesis. Methionine adenosyltransferase is an enzyme which catalyzes the synthesis of SAM from methionine and ATP, while MAT catalyzes the only reaction that produces the major methyl donor. These two enzymes are detected in dinoflagellates [33,34]. In our study, MAT was up-regulated at the initial toxin biosynthesis stage (T28), suggesting that more methionine and ATP were utilized to synthesize SAM, the direct precursor of PST biosynthesis in the first step. Up-Regulation of MAT at T28 indicated that more methyl donor was produced for toxin biosynthesis in step 5. ADH is encoded by the *sxt*U gene and participates in the eighth step of PST synthesis in cyanobacteria. This enzyme is mostly NAD(P)H-dependent, for the reduction of aldehydes and ketones to an alcohol group in the cells. This enzyme is also identified in dinoflagellates [35,36]. The obvious up-regulation of ADH at T34 might provide more electrons for PST biosynthesis. PPi is a ubiquitous enzyme that catalyzes the conversion of one molecule of pyrophosphate to two phosphate (Pi) ions [37]. This process is coupled with fatty acid degradation which is catalyzed by the enzyme acyl-CoA synthetase to produce acyl-CoA, an important intermediate for PST biosynthesis. Moreover, this reaction is highly exergonic and therefore greatly increases the energetic favorability of the reaction system when coupled with a typically less-favorable reaction. The significant up-regulation of this enzyme at T34 indicated that PST biosynthesis might be a highly exergonic process and also that there is a high requirement for acyl-CoA. FNR catalyzes reduced ferredoxin, NADP+ and H+ to oxidized ferredoxin and NADPH. In cyanobacteria, this enzyme is encoded by the *sxt*W gene and provides H^+^ for toxin synthesis in step 5 and 8 of the PST biosynthesis pathway. This enzyme is identified in *A. catenella* at the transcriptional level [32,33]. The up-regulation of this enzyme at the toxin synthesizing stage (T34) might increase the production of H^+^ for PST biosynthesis in *A. catenella*. The enzyme OTC, also called ornithine transcarbamoylase, is confirmed in *A. fundyense *and *A. minutum *at the transcriptional level [15]. It was encoded by *Sxt*I which catalyzed the reaction between carbamoylphosphate (CP) and ornithine to form citrulline and Pi. In step 10, *Sxt*I in conjunction with *Sxt*J and *Sxt*IK catalyzed a carbamoyltransfer form of CP onto the free hydroxyl at C-13, forming saxitoxin (STX). The up-regulation of this enzyme at T34 indicated that more citrulline and Pi were donated for STX production.

In enzymology, ADI is an enzyme catalyzing l-arginine and H_2_O to l-citrulline and NH_3_, which acts on carbon-nitrogen bonds other than peptide bonds, specifically in linear amidines. Arginine is known as one of the primary precursors of PST in both cyanobacteria and dinoflagellates. In this study, the expression of ADI was depressed at the toxin biosynthesizing stage (T34), indicating that more arginine was invested in toxin biosynthesis. SULT, an important modification enzyme involved in PST conversion, has been found in several dinoflagellate species, and can transfer a sulfate group to N-21 in the carbamoyl group of GTX2/3 or STX and produce various STX derivatives [14,29,38,39,40]. In cyanobacteria, this enzyme is encoded by the *sxt*N gene and participates in the transfer of a sulfate group to N-21 or O-21. The up-regulation of this enzyme at T34 indicated that active conversion of GTX2/3 to C1/2 toxin was occurring. The predominant composition of the C1/2 toxin (about 90% of the total toxin) with only a trace amount of GTX2/3 toxin in *A. catenella* supported this postulation [28].

**Table 1:** Variations of nine proteins putatively involved in toxin biosynthesis in *A. catenella* at different toxin biosynthesis phases.

Spot id	Accession number	Protein score	Protein score CI%	Peptide count	MW/*p*I	Protein description	Function	T28 *vs.* T24		T34 *vs*. T24	
								Foldchange	*P*-Value	Fold change	*P*-Value
***Metabolism***											
31	46909371	145	100	4	34.39/5.81	Methionine adenosyltransferase (MAT), (*Nucula proxima*)	catalyses the synthesis of *S*-adenosylmethionine (SAM) from methionine and ATP	1.26	0.450	0.40	0.043
32	158524698	149	100	3	35.30/5.59	Methionine adenosyltransferase (MAT), (*Terebratulina retusa*)		1.89	0.173	0.47	0.033
33	71370920	202	100	3	35.08/6.82	Methionine adenosyltransferase (MAT), partial (*Haliotis rufescens*)		1.15	0.767	0.40	0.034
34	225685869	121	100	3	45.52/5.54	Methionine *S*-adenosyltransferase (MAT), (*Thalassionema nitzschioides*)		0.92	0.942	0.46	0.042
36	225685865	92	99.248	2	51.08/5.73	methionine *S*-adenosyltransferase (MAT), (*Detonula confervacea*)		2.50	0.007	0.18	0.091
77	211939908	120	99.999	9	27.97/5.74	*S*-adenosylhomocysteinase (SAH), (*Amphidinium carteriae*)	an intermediate in the synthesis of cysteine and formed by the demethylation of SAM	2.89	0.001	0.86	0.905
76	211939908	150	100	8	27.97/5.74	Adenosylhomocysteinase (AdoHcy), (*Amphidinium carteriae*)	an enzyme that converts SAH to homocysteine and adenosine	2.20	0.003	0.49	0.117
79	211939908	116	99.997	7	27.97/5.74			3.32	0.003	0.88	0.985
7	58613455	389	100	2	28.67/4.78	chloroplast ferredoxin-NAD + reductase (FNRs), (*Heterocapsa triquetra*)	ferredoxin-NADP reductase type 1 family; Oxidation reduction	0.13	0.007	1.19	0.641
40	170723385	296	100	15	38.10/5.92	ornithine carbamoyltransferase (OTC), (*Pseudomonas putida *W619)	Cellular amino acid metabolic process; involved in arginine (Arg) biosynthesis	0.45	0.088	1.61	0.003
55	26987276	87	97.728	2	19.18/4.77	inorganic pyrophosphatase (PPi), (*Pseudomonas putida *KT2440)	Phosphate metabolic process; catalyzes the conversion of pyrophosphate to phosphate ions	1.00	1.000	11.55	0.003
78	148546281	660	100	18	46.73/5.66	arginine deiminase (ADI), (*Pseudomonas putida *F1)	participates in arginine and proline metabolism	3.30	<0.001	0.20	0.008
94	115901552	122	97.645	1	35.22/4.70	similar to sulfotransferase (SULT), (*Strongylocentrotus purpuratus*)	sulfotransferase activity	0.44	0.043	1.24	0.459
**Oxidative stress response**											
95	26990544	110	99.989	6	35.89/5.61	alcohol dehydrogenase (ADH), (*Pseudomonas putida *KT2440)	Oxidation reduction; zinc ion binding	0.45	0.871	7.63	0.001

**Figure 5:**
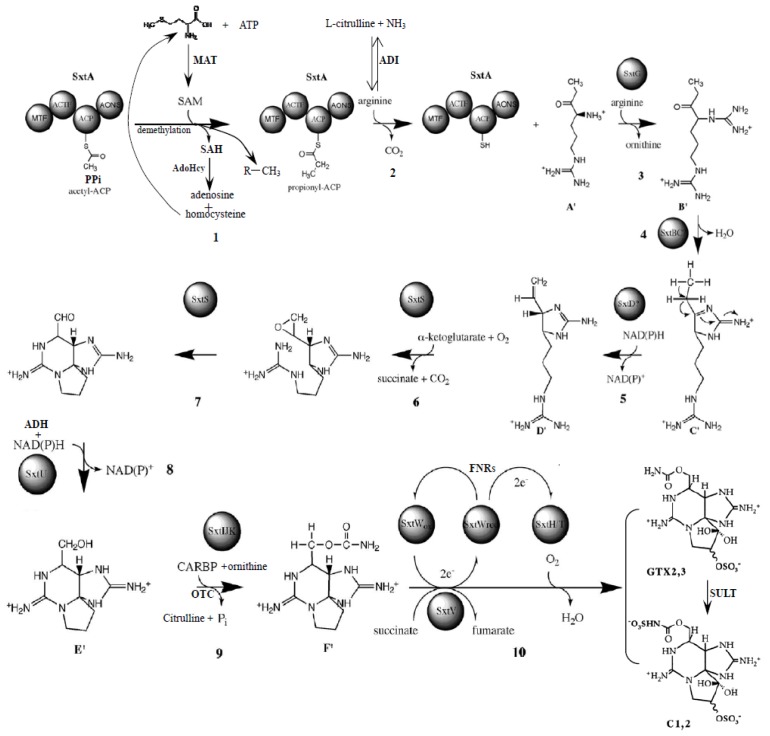
Proposed toxin biosynthesis pathway in *A. catenella* (modified from Mihali *et al*., 2009 [19]). PPi: inorganic pyrophosphatase; MAT: methionine *S*-adenosyltransferase; SAH: *S*-adenosylhomocysteinase; AdoHcy: adenosylhomocysteinase; OTC: ornithine carbamoyltrans-ferase; SULT: similar to sulfotransferase; ADH: alcohol dehydrogenase; FNR: chloroplast ferredoxin-NADP+ reductase; ADI: arginine deiminase.

Furthermore, we analyzed the interactions of all the proteins identified using KEGG software and found that the above nine proteins were linked to each other through the definite or assumptive proteins, the detailed interpretation of the interaction network is shown in Supplemental file (Supplemental Figure S1). Taking MAT3 (methionine adenosyltransferase) as an example: Using protein-protein and chemical-protein interactions, MAT3 interacted with SAH and adenosylhomocysteinase (ACHY) through a series of chemical reactions, *i.e.*, MAT3 could catalyze the synthesis of SAM from methionine and ATP, SAHH2 could convert SAH to homocysteine and adenosine, SAH was an intermediate in the synthesis of cysteine and formed by the demethylation of SAM. MAT3 interacted with AVPL1 (inorganic pyrophosphatase) through two functional proteins, adenosine diphosphate (ADP) and SAM. MAT3 interacted with SULT and AT4G22110 (alcohol dehydrogenase) via several predicted proteins and intermediates, *i.e.*, malonate, acetaldehyde, SAH and SAHH. MAT3 interacted with AT5G47435 (formyltetrahydrofolate deformylase) via selenomethionine. MAT3 interacted with EMB1873 (arginine deiminase) through AT5G15950 (adenosylmethionine decarboxylase family protein) and ADC2 (arginine decarboxylase). MAT3 interacted with PEIF (chloroplast ferredoxin-NADP+ reductase) by EMB1873 (arginine deiminase). MAT3 interacted with OTC via ornithine, ammonia and SAM. Thus it could be seen that the nine proteins linked to each other through the complex interaction network. This implied that the above nine proteins may complete a biological process together in collaborative or mutually promotive ways. Related to the functions of these proteins in the PST biosynthesis pathway in toxic cyanobacteria, we postulated that these nine proteins might be involved in toxin biosynthesis and participate in the different toxin biosynthesis steps in *A. catenella* (Figure 5).

## 3. Experimental Section

### 3.1. Organism and Culture Conditions

*A. catenella* was provided by the Collection Center of Marine Bacteria and Algae, Xiamen University, China. The culture was routinely maintained in K culture medium [41] at 20 °C under a 14:10 h light: Dark photoperiod at a light intensity of approximately 100 μE/m^2^·s provided by fluorescent lamps.

### 3.2. Synchronization of *A. catenella*

Cultures of *A. catenella* were synchronized using a dark induced method. Low density (2000 ± 60 cells/mL), exponential growing cultures of *A. catenella* were grown in the original light-dark cycle (14:10 h, light/dark). When the cells reached high density (8000 ± 202 cells/mL), synchronization of cells was achieved by maintaining the exponential growing cells in continuous darkness for 36 h. Subsequently, the vigorous synchronized cells were filtered using 10 μm filter meshes and rinsed three times with autoclaved seawater, then transferred into the original L/D cycle with an initial cell density of 3500 ± 180 cells/mL. After completion of the dark cycle on the second day after inoculation, time zero point (T0 of the experiment) was designated, and samples for cell count, flow cytometry, toxin content and proteome analysis were taken every 2 h from T0 to T38. 

### 3.3. Cell Count

Cell density was monitored every 2 h. Three 1 mL samples were collected in 1.5 mL Eppendorf tubes and fixed with 30 μL of lugol’s iodine solution. Cell count was conducted manually under a light microscope.

### 3.4. Cell Cycle Defined

Approximately 5 × 10^5^ cells were prepared for flow cytometric analysis as previous described by Olson *et al*. [42]. Cells were harvested using centrifugation at 2000× *g* for 5 min, rinsed twice with sterilized seawater to avoid any carry-over of culture medium and fixed with 1 mL of 70% cold ethanol to extract their cellular pigments. Prior to flow cytometric analysis, the fixed cells were stained with a base intercalating dye, 10 μg/mL PI (propidium iodide) in phosphate buffered saline containing 40 units/mL RNase and 0.3% TritonX-100. Cell cycle analysis was performed on an Epics XL flow cytometer (Beckman Coulter, USA) with a 5-W argon laser having a 488 nm excitation wavelength. The 635 nm emission wavelength was monitored for PI emission. Histograms of relative DNA content were analyzed using MultiCycle software (Beckman Coulter) to quantify the percentage of cells in each of the stages (G1, S, G2 + M) of the cell cycle. All reagents were obtained from Sigma unless otherwise mentioned.

### 3.5. Toxin Analysis

Samples for toxin analysis were taken every 2 h from T0 through T38. About 2.5–5 × 10^5^ cells were collected in three 50 mL tubes by centrifugation for 10 min (3000× *g*, 20 °C). After being washed twice with autoclaved seawater, the pellets were transferred into centrifuge tubes with 0.5 mL of 0.5 M acetic acid and sonicated in an ice bath for 5 min with short pulses of 5 s (model 450, Branson Ultrasonics, Danbury, CT, USA). Subsequently, the extracts were centrifuged for 30 min (10,000× *g*, 20 °C). The supernatants were passed through a C18 cartridge following the manufacturer’s protocol. The eluents were collected and then spun in a Millipore 10,000 MW cutoff filter at 4000× *g* for 5 min. The purified cell extracts, approximately 200 μL from each, were loaded into separate auto sampler vials, and analyzed for PST derivatives using HPLC with a post column system (HP1100; Agilent, Santa Clara, CA, USA) using the three step isocratic elution method of Oshima *et al*. [43]. The toxin standards (C1,2, GTX1-5, STX, neo STX, dc STX) were obtained from the National Research Council Canada. 

### 3.6. Protein Extraction and Quantification

Three samples collected at T24, T28 and T34, representing the non-toxin synthesis, initial toxin synthesis and toxin synthesizing stages of *A. catenella*, were selected for 2-DE analysis. Approximately 1 × 10^7^ cells for each sample were collected by centrifugation at 2000× *g* for 5 min at 20 °C. The pellet was subsequently transferred to a 1.5 mL centrifuge tube, rinsed twice with autoclaved seawater to avoid any carry-over of culture medium and extracellular proteins. 1 mL Trizol reagent was added to the cell pellets which were then sonicated using an ultrasonic disrupter (Model 450, Branson Ultrasonics, Danbury, CT, USA). Subsequently, 200 μL of chloroform was added to the cell lysate and the mixture was shaken vigorously for 15 s. The mixture was allowed to stand for 5 min at room temperature before centrifugation at 12,000× *g* for 15 min at 4 °C. The pale-yellow or colorless upper layer was removed. 300 μL of ethanol was added to re-suspend the bottom layer and the mixture was centrifuged at 2000× *g* for 5 min at 4 °C. The supernatant was transferred to a new tube and 1.5 mL of isopropanol was added. The mixture was allowed to stand for at least 20 min for protein precipitation at room temperature, then centrifuged at 14,000× *g* for 10 min at 4 °C. The pellet was washed with 95% ethanol twice and allowed to air dry. Finally, 150 μL of rehydration buffer was added to dissolve the protein pellet.

Protein quantification was performed using the PlusOneTM 2D Quant kit (GE Healthcare Life Sciences). 

### 3.7. 2-DE Analysis

Typically, a 340 μL sample containing 100 μg of protein (for silver staining and protein identification) in rehydration buffer containing 7 M urea, 2 Mthiourea, 4% CHAPS, 0.2% DTT and 1.5 μL of pH 4–7 IPG buffer was used to rehydrate 18 cm pH 4–7 IPG strips (Bio-Rad, USA) for 13 h. After rehydration, IEF was performed using the IPGphor 3 (GE Life Science, USA). Voltage control was performed using the following schedule: 2 h at 100 V, 2 h at 200 V, 1 h at 500 V, 2 h at 1000 V, 2 h at 4000 V, 2 h at 8000 V, and then until the total Vhrs reached 50,000. After the first dimension run, each strip was equilibrated with about 10 mL of equilibration buffer (50 mM Tris, pH 8.8, 6 M urea, 30% glycerol, 2% SDS, 1% DTT, and trace amounts of bromophenol blue) for 20 min. The gel strip was then equilibrated in fresh equilibration buffer containing 1% iodoacetamine (instead of DTT) for a further 20 min. The second-dimension SDS/PAGE was performed using 12.5% polyarylamide gel, running at a constant current of 25 mA/gel until the bromophenol blue dye reached the end of the gel. After electrophoresis, the gel was stained with silver mainly following the method of Wang *et al*. [24]. After staining, the gels were scanned using a Perfection Gel documentation system on a GS-670 Imaging Densitometer from Bio Rad and 2-DE electrophoretogram matching software. The images were analyzed using ImageMaster 2D 5.0 Platinum as described in the user manual.

Each sample was analyzed in triplicate using 2-DE and only protein spots consistently present in all three gels were considered. An at least 2-fold difference (*P* < 0.05) in spot optical density was taken to indicate differentially expressed protein spots. The gels shown are representatives of the triplicates.

### 3.8. MALDI-TOF-TOF MS Analysis

Differentially expressed protein spots were manually excised from the silver stained gels and transferred to a 96 well plate (Eppendorf, Germany). Each spot was washed twice in milli-Q water and destained in a destaining buffer (0.16 g sodium thiosulfate and 0.1 g potassium ferricyanide in 10 mL MilliQ water), then washed with Milli-Q water at least five times (400 μL/well). Subsequently, the gels were dehydrated using 100% acetonitrile (CAN, 200 μL/well) and dried at room temperature for 10–15 min, before being digested in gel with trypsin (10 ng/μL in 25 mM ammonium bicarbonate) for 16 h at 37 °C (or 50 °C for 2 h). Protein identification was conducted using an AB SCIEX MALDI TOF-TOF™ 5800 Analyzer (AB SCIEX, Foster City, CA, USA) equipped with a neodymium: yttrium-aluminum-garnet laser (laser wavelength 349 nm). The TOF/TOF calibration mixtures (AB SCIEX) were used to calibrate the spectrum to a mass tolerance within 150 ppm. For the MS mode, peptide mass maps were acquired in positive reflection mode, and the 850–4000 *m*/*z* mass range was used with 1000 laser shots per spectrum. The PMF peak detection criteria used were: minimum signal/noise (S/N) of 10, local noise window width mass/charge (*m*/*z*) of 250 and minimum full-width half-maximum (bins) of 2.9. A maximum of 20 precursors per spot with a minimum S/N ratio of 50 were selected for MS/MS analysis using ambient air as collision gas with a medium pressure of 10–6 Torr. The contaminant *m*/*z* peaks originating from human keratin, trypsin auto-digestion, or matrix were excluded for MS/MS analysis. An energy of 1 kV was used for collision-induced dissociation, and 2000 acquisitions were accumulated for each MS/MS spectrum. The peak detection criteria used were: minimum S/N of 3, local noise window width (*m*/*z*) of 200 and minimum full-width half-maximum (bins) of 2.9. A combined MS and MS/MS search was performed against the NCBInr database with no taxonomic restriction (updated December, 2010, containing 4,607,655 entries). All database searching was fulfilled using the GPS Explorer™ software (version 3.6, AB SCIEX) running a mascot search algorithm (v2.2, Matrix Science, London, UK) for protein identification. Results with CI% values greater than 95% were considered to be a positive identification. The identified proteins were then matched to specific processes or functions by searching Gene Ontology.

### 3.9. Protein Interaction Network Analysis

The differentially expressed proteins were further analyzed for their association with network pathways using STITCH which aims to integrate the databases of biological pathways, drug-target relationships, binding affinities and relevant interactions [44]. It is a resource to explore known and predicted interactions of chemicals and proteins. Chemicals are linked to other chemicals and proteins by evidence derived from experiments, databases and the literature. To explore the interactions of multiple proteins, enter a mixture of protein and chemical names (one per line) in the search box to the left. Select “auto-detect” as organism. Then click “GO!”, it will be taken to a network containing both proteins and related chemicals.

### 3.10. Statistical Analysis

Each sample was run in triplicate and the abundance of each protein spot was expressed as mean value (*n* = 3) ± standard deviation (SD). Statistical analysis was performed using one-way ANOVA (IBM SPSS statistics 19) to evaluate whether the mean value was significantly different among the three time points (T24, T28 and T34). Before running one-way ANOVA test, data were log transformed to meet ANOVA assumptions of normality and variance homoscedasticity. Only those proteins with *P*-value < 0.05 were considered statistically significant.

## 4. Conclusions

This study, to our knowledge, for the first time, compared the protein profiles of the PST-producing dinoflagellate, *A. catenella*, at different toxin biosynthesis stages, and identified 53 differentially expressed proteins. These proteins were involved in various biological processes, nine of which might be involved in the PST biosynthesis of *A. catenella *based on their functions in the PST-producing cyanobacteria combined with their interaction network. In future research, we intend to compare transcriptomic profiles in order to identify the unique genes as well as the highly expressed genes of *A. catenella* at different toxin biosynthesis stages. Meanwhile, we will use the quantitative proteomic approach, *i.e.*, 2-D DIGE to compare protein profiles at different toxin biosynthesis stages. These should help to reveal the toxin biosynthesis mechanism and pathway in dinoflagellates.

## Supplementary Files

Supplementary File 1 Supplementary Figure (PDF, 320 KB)

Supplementary File 2 Supplementary Table (XLS, 67 KB)
