# Supplementary material for: Comparative Proteomic Analysis Reveals Proteins Putatively Involved in Toxin Biosynthesis in the Marine Dinoflagellate Alexandrium catenella
Source: Mar Drugs. 2013 Jan 22;11(1):213–32. doi: 10.3390/md11010213 (PMC3564168; doi:10.3390/md11010213)
Supplement: Supplementary File 1 — Supplementary Figure (PDF, 320 KB) [file marinedrugs-11-00213-s001.pdf]

Supplementary Figure

Figure S1. Interpretation of protein interaction network on nine proteins putatively involved in toxin biosynthesis in *A. catenella* (http://stitch.embl.de; organism: *Arabidopsis thaliana*). Stronger interactions are represented by bold lines. Protein-protein interactions are shown in blue, chemical-protein interactions in green and chemical-chemical interactions in red.

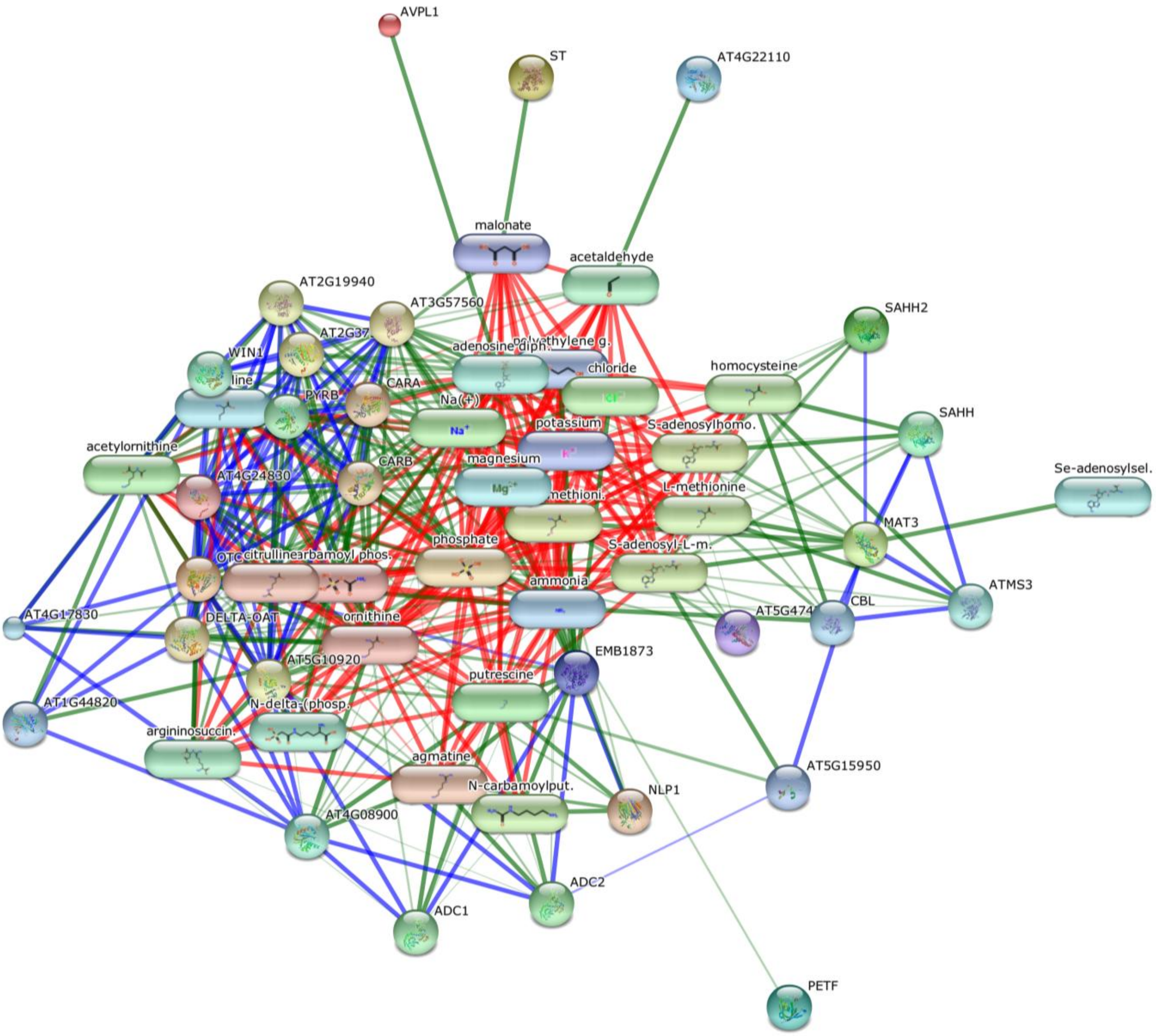

9 Proteins We Input

- <u>AVPL1</u> vacuolar-type H+-translocating inorganic pyrophosphatase, putative; vacuolar-type H+-translocating inorganic pyrophosphatase, putative; FUNCTIONS IN- inorganic diphosphatase activity, hydrogen-translocating pyrophosphatase activity; INVOLVED IN- proton transport; LOCATED IN- vacuole; EXPRESSED IN- 22 plant structures; EXPRESSED DURING- 13 growth stages; CONTAINS InterPro DOMAIN/s- Inorganic H+ pyrophosphatase (InterPro-IPR004131); BEST Arabidopsis thaliana protein match is- AVP2 (ARABIDOPSIS VACUOLAR H+-PYROPHOSPHATASE 2); hydrogen-translocating pyrophosphatase (TAIR-AT1G78920.2); Has [...] (802 aa)

OTC	OTC (ORNITHINE CARBAMOYLTRANSFERASE); amino acid binding/carboxyl- or carbamoyltransferase/ornithine carbamoyltransferase; ORNITHINE CARBAMOYLTRANSFERASE (OTC); FUNCTIONS IN- amino acid binding, ornithine carbamoyltransferase activity, carboxyl- or carbamoyltransferase activity; INVOLVED IN- amino acid metabolic process; LOCATED IN- chloroplast, chloroplast stroma; EXPRESSED IN- 23 plant structures; EXPRESSED DURING- 13 growth stages; CONTAINS InterPro DOMAIN/s- Aspartate/ornithine carbamoyltransferase, carbamoyl-*P* binding (InterPro-IPR006132), Aspartate/ornithine carbamoyltransfera [...] (375 aa)
ST	ST; brassinosteroid sulfotransferase/sulfotransferase; Encodes a brassinosteroid sulfotransferase. *In vitro* experiements show that this enzyme has a preference for 24-epibrassinosteroids, particularly 24-epicathasterone, but does not act on castasterone and brassinolide. It is differentially expressed during development, being more abundant in young seedlings and actively growing cell cultures. Expression is induced in response to salicylic acid and methyl jasmonate and bacterial pathogens. (326 aa)
MAT3	MAT3 (methionine adenosyltransferase 3); copper ion binding/methionine adenosyltransferase; methionine adenosyltransferase 3 (MAT3); FUNCTIONS IN- copper ion binding, methionine adenosyltransferase activity; INVOLVED IN- one-carbon compound metabolic process, *S*-adenosylmethionine biosynthetic process; LOCATED IN- plasma membrane; EXPRESSED IN- 27 plant structures; EXPRESSED DURING- 16 growth stages; CONTAINS InterPro DOMAIN/s- *S*-adenosylmethionine synthetase (InterPro-IPR002133); BEST Arabidopsis thaliana protein match is- MTO3 (METHIONINE OVER-ACCUMULATOR 3); methionine adenosyltran [...] (390 aa)
SAHH2	SAHH2 (*S*-ADENOSYL-L-HOMOCYSTEINE (SAH) HYDROLASE 2); adenosylhomocysteinase/binding/catalytic; *S*-ADENOSYL-L-HOMOCYSTEINE (SAH) HYDROLASE 2 (SAHH2); FUNCTIONS IN- adenosylhomocysteinase activity, binding, catalytic activity; INVOLVED IN- one-carbon compound metabolic process, metabolic process; LOCATED IN- plasma membrane, vacuole, membrane; EXPRESSED IN- 32 plant structures; EXPRESSED DURING- 15 growth stages; CONTAINS InterPro DOMAIN/s- *S*-adenosyl-L-homocysteine hydrolase (InterPro-IPR000043), NAD(P)-binding (InterPro-IPR016040), *S*-adenosyl-L-homocysteine hydrolase, NAD binding (In [...] (485 aa)
SAHH	MEE58 (MATERNAL EFFECT EMBRYO ARREST 58); adenosylhomocysteinase; Encodes a *S*-adenosyl-L-homocysteine hydrolase required for DNA methylation-dependent gene silencing.; Adenosylhomocysteine is a competitive inhibitor of *S*-adenosyl-L-methinine-dependent methyl transferase reactions; therefore adenosylhomocysteinase may play a key role in the control of methylations via regulation of the intracellular concentration of adenosylhomocysteine. Required for DNA methylation-dependent gene silencing (485 aa)
PETF	ferredoxin family protein; ferredoxin family protein; FUNCTIONS IN- electron carrier activity, iron-sulfur cluster binding, 2 iron, 2 sulfur cluster binding; INVOLVED IN- electron transport chain; LOCATED IN- chloroplast; EXPRESSED IN- 23 plant structures; EXPRESSED DURING- 13 growth stages;

CONTAINS InterPro DOMAIN/s- Ferredoxin (InterPro-IPR001041), Ferredoxin [2Fe-2S], plant (InterPro-IPR010241), Beta-grasp fold, ferredoxin-type (InterPro-IPR012675); BEST Arabidopsis thaliana protein match is- ATFD3 (ferredoxin 3); 2 iron, 2 sulfur cluster binding/electron carrier/iron-sulfur clu [...] (154 aa)

- AT4G22110 alcohol dehydrogenase, putative; alcohol dehydrogenase, putative; FUNCTIONS IN- oxidoreductase activity, binding, zinc ion binding, catalytic activity; INVOLVED IN- oxidation reduction, metabolic process; LOCATED IN- cellular_component unknown; EXPRESSED IN- root; CONTAINS InterPro DOMAIN/s- GroES-like (InterPro-IPR011032), NAD(P)-binding (InterPro-IPR016040), Alcohol dehydrogenase GroES-like (InterPro-IPR013154), Alcohol dehydrogenase, zinc-containing, conserved site (InterPro-IPR002328), Alcohol dehydrogenase, zinc-binding (InterPro-IPR013149), Alcohol dehydrogenase superfamily, zinc [...] (389 aa)
- EMB1873 EMB1873 (EMBRYO DEFECTIVE 1873); agmatine deiminase; EMBRYO DEFECTIVE 1873 (EMB1873); FUNCTIONS IN- agmatine deiminase activity; INVOLVED IN- embryonic development ending in seed dormancy, polyamine biosynthetic process; LOCATED IN- cellular_component unknown; EXPRESSED IN- 22 plant structures; EXPRESSED DURING- 13 growth stages; CONTAINS InterPro DOMAIN/s- Agmatine deiminase (InterPro-IPR017754), Peptidyl-arginine deiminase, Porphyromonas-type (InterPro-IPR007466); Has 1681 Blast hits to 1679 proteins in 426 species—Archae-10; Bacteria-903; Metazoa-6; Fungi-22; Plants-28; V [...] (383 aa)

Predicted Functional Partners

**NAME**	**DESCRIPTION**	**score**
AT4G24830	arginosuccinate synthase family; arginosuccinate synthase family; FUNCTIONS IN- argininosuccina [...] (494 aa)	0.999
carbamoyl phos.	carbamoyl phosphate; Carbamoyl phosphate is an anion of biochemical significance. In land-dwell [...] (141.0 g/mol)	0.999
ornithine	Ornithine is an amino acid that plays a role in the urea cycle. (132.2 g/mol)	0.998
citrulline	The organic compound citrulline is an -amino acid. Its name is derived from citrullus, the Lati [...] (175.2 g/mol)	0.997
agmatine	Decarboxylated arginine, isolated from several plant and animal sources, e.g., pollen, ergot, h [...] (130.2 g/mol)	0.996
NLP1	NLP1 (NITRILASE-LIKE PROTEIN 1); *N*-carbamoylputrescine amidase/hydrolase, acting on carbon-nit [...] (326 aa)	0.996
CARA	CARA (CARBAMOYL PHOSPHATE SYNTHETASE A); carbamoyl-phosphate synthase (glutamine-hydrolyzing)/[...] (430 aa)	0.993
CARB	CARB (CARBAMOYL PHOSPHATE SYNTHETASE B); ATP binding/carbamoyl-phosphate synthase/catalytic; [...] (1187 aa)	0.992

phosphate	A phosphate, an inorganic chemical, is a salt of phosphoric acid. In organic chemistry, a phosp [...] (97.0 g/mol)	0.991
DELTA-OAT	delta-OAT; ornithine-oxo-acid transaminase; ornithine delta-aminotransferase (475 aa)	0.988
AT3G57560	aspartate/glutamate/uridylate kinase family protein; encodes a *N*-acetylglutamate kinase, involv [...] (347 aa)	0.983
AT2G37500	arginine biosynthesis protein ArgJ family; arginine biosynthesis protein ArgJ family; FUNCTIONS [...] (468 aa)	0.982
AT5G10920	argininosuccinate lyase, putative/arginosuccinase, putative; argininosuccinate lyase, putativ [...] (517 aa)	0.981
AT2G19940	*N*-acetyl-gamma-glutamyl-phosphate reductase/NAD or NADH binding/binding/catalytic/oxidore [...] (401 aa)	0.979
selenomethioni.	selenomethionine; A selenium (Se) analogue of methionine in which sulfur is replaced with the t [...] (196.1 g/mol)	0.979
S-adenosylhomo.	*S*-adenosylhomocysteine; *S*-Adenosyl-L-homocysteine (SAH) is an amino acid derivative used in sev [...] (384.4 g/mol)	0.978
S-adenosyl-L-m.	*S*-adenosyl-L-methionine; *S*-Adenosyl methionine (SAM, SAMe, SAM-e) is a common co-substrate invo [...] (399.4 g/mol)	0.977
L-methionine	A synthetic amino acid radiolabeled with carbon-11. Acting as a methyl donor, methionine C 11 i [...] (149.2 g/mol)	0.973
N-carbamoylput.	*N*-carbamoylputrescine (131.2 g/mol)	0.971
homocysteine	A thiol-containing amino acid formed by a demethylation of methionine. (135.2 g/mol)	0.966
chloride	The chloride ion is formed when the element chlorine, a halogen, picks up one electron to form [...] (35.5 g/mol)	0.965
Na(+)	Sodium hydride is the chemical compound with the empirical formula NaH. It is primarily used as [...] (23.0 g/mol)	0.965
acetylornithine	acetylornithine (174.2 g/mol)	0.962
putrescine	Putrescine (sometimes spelled putrescin) is a foul-smelling organic chemical compound NH2(CH2)4 [...] (88.2 g/mol)	0.958
ADC2	ADC2 (ARGININE DECARBOXYLASE 2); arginine decarboxylase; encodes a arginine decarboxylase (ADC) [...] (711 aa)	0.947
ADC1	ADC1 (ARGININE DECARBOXYLASE 1); arginine decarboxylase; encodes a arginine decarboxylase (ADC) [...] (702 aa)	0.945
PYRB	aspartate carabmoyltransferase, chloroplast/aspartate transcarbamylase/ATCase (PYRB); encod [...] (390 aa)	0.942
acetaldehyde	Acetaldehyde (systematically ethanal) is an organic chemical compound with the formula CH3CHO o [...] (44.1 g/mol)	0.941
argininosuccin.	argininosuccinate (290.3 g/mol)	0.941
WIN1	WIN1 (HOPW1-1-INTERACTING 1); *N*2-acetyl-L-ornithine-2-oxoglutarate 5-aminotransferase/catalyti [...] (457 aa)	0.933
N-delta-(phosp.	*N*-delta-(phosphonoacetyl)-L-ornithine (254.2 g/mol)	0.930

AT4G08900	arginase; Encodes an arginase, likely to be involved in polyamine biosynthesis in pollen. (342 aa)	0.922
adenosine diph.	adenosine diphosphate; Adenosine diphosphate, abbreviated ADP, is a nucleotide. It is an ester [...] (427.2 g/mol)	0.921
ATMS3	ATMS3 (methionine synthase 3); 5-methyltetrahydropteroyltriglutamate-homocysteine S-methyltrans [...] (812 aa)	0.917
Se-adenosylsel.	Se-adenosylselenomethionine (445.3 g/mol)	0.911
magnesium	Magnesium hydride is the chemical compound MgH2. It contains 7.66% by weight of hydrogen and ha [...] (24.3 g/mol)	0.907
norvaline	Differs from valine in being 1 carbon longer instead of branched. (117.1 g/mol)	0.906
AT4G17830	peptidase M20/M25/M40 family protein; peptidase M20/M25/M40 family protein; FUNCTIONS IN- metal [...] (440 aa)	0.904
CBL	CBL (cystathionine beta-lyase); cystathionine beta-lyase; Encodes second enzyme in the methioni [...] (464 aa)	0.904
ammonia	Ammonia is a compound of nitrogen and hydrogen with the formula NH3. It is a colourless gas wit [...] (17.0 g/mol)	0.903
AT1G44820	aminoacylase, putative/*N*-acyl-L-amino-acid amidohydrolase, putative; aminoacylase, putative/[...] (438 aa)	0.900
AT5G15950	adenosylmethionine decarboxylase family protein; adenosylmethionine decarboxylase family protei [...] (362 aa)	0.900
polyethylene g.	polyethylene glycol; Ethylene glycol (IUPAC name: ethan-1, 2-diol) is an organic compound widely [...] (62.1 g/mol)	0.900
potassium	An element with atomic symbol K, atomic number 19, and atomic weight 39.10. (39.1 g/mol)	0.900
malonate	Malonic acid (IUPAC systematic name: propanedioic acid) is a dicarboxylic acid with structure C [...] (104.1 g/mol)	0.900
